# Post-transcriptional inactivation of matrix metalloproteinase-12 after focal cerebral ischemia attenuates brain damage

**DOI:** 10.1038/srep09504

**Published:** 2015-05-08

**Authors:** Bharath Chelluboina, Aditi Warhekar, Matt Dillard, Jeffrey D. Klopfenstein, David M. Pinson, David Z. Wang, Krishna Kumar Veeravalli

**Affiliations:** 1 Department of Cancer Biology and Pharmacology, University of Illinois College of Medicine at Peoria, Peoria, IL, USA; 2 Department of Neurosurgery, University of Illinois College of Medicine at Peoria, Peoria, IL, USA; 3 Department of Pathology, University of Illinois College of Medicine at Peoria, Peoria, IL, USA; 4 Department of Neurology, University of Illinois College of Medicine at Peoria, Peoria, IL, USA; 5 Comprehensive Stroke Center, Illinois Neurological Institue, Peoria, IL, USA

## Abstract

This study highlights the possible pathological role of MMP-12 in the context of ischemic stroke. Male rats were subjected to a two-hour middle cerebral artery occlusion (MCAO) procedure. MMP-12 shRNA expressing plasmid formulation was administered to these rats twenty-four hours after reperfusion. The results showed a predominant upregulation of MMP-12 (approximately 47, 58, 143, and 265 folds on days 1, 3, 5, 7 post-ischemia, respectively) in MCAO subjected rats. MMP-12 expression was localized to neurons, oligodendrocytes and microglia, but not astrocytes. Transcriptional inactivation of MMP-12 significantly reduced the infarct size. The percent infarct size was reduced from 62.87 ± 4.13 to 34.67 ± 5.39 after MMP-12 knockdown compared to untreated MCAO subjected rats. Expression of myelin basic protein was increased, and activity of MMP-9 was reduced in ischemic rat brains after MMP-12 knockdown. Furthermore, a significant reduction in the extent of apoptosis was noticed after MMP-12 knockdown. TNFα expression in the ipsilateral regions of MCAO-subjected rats was reduced after MMP-12 knockdown in addition to the reduced protein expression of apoptotic molecules that are downstream to TNFα signaling. Specific knockdown of MMP-12 after focal cerebral ischemia offers neuroprotection that could be mediated via reduced MMP-9 activation and myelin degradation as well as inhibition of apoptosis.

Globally, fifteen million people suffer from a stroke each year and five million stroke patients die. Another five million are left permanently disabled^1^. Despite decades of work, no clinically effective pharmacotherapies exist to facilitate cellular functional recovery after a stroke. Although thrombolysis with tissue plasminogen activator (tPA) is a stroke therapy approved by the FDA, its efficacy may be limited by neurotoxic side effects^2^,^3^. At least some of the neurotoxic properties of tPA may involve the induction of matrix metalloproteinases (MMPs)^4^. Additionally, reperfusion of the ischemic tissue with thrombolytic therapy, which involves the reintroduction of oxygenated blood into the ischemic region, enhances the release of proteases such as MMPs, which are mainly produced by vascular smooth muscle cells, monocytes and endothelial cells. MMPs are a large family of proteolytic enzymes involved in inflammation, wound healing, and other pathological processes after neurological injury. Available data suggests that MMPs have a deleterious role in stroke. Rapid upregulation of MMPs has been reported at 24–48 h after cerebral ischemia^5^,^6^,^7^. MMPs degrade or proteolytically modify essentially all of the extracellular matrix (ECM) components, including collagens, laminin, and proteoglycans. By degrading neurovascular matrix, MMPs promote injury of the blood-brain-barrier (BBB), edema, and hemorrhage^4^,^8^,^9^. Pharmacological inhibition of MMPs reduced brain damage and edema^10^,^11^. MMPs also trigger brain cell death by disrupting cell-matrix signaling and homeostasis^12^,^13^.

MMP-12 has the ability to activate other MMPs such as pro-MMP-2 and pro-MMP-3, which, in turn, can activate pro-MMP-1 and pro-MMP-9^14^. MMP-2 and MMP-9 destroy the capillary tight junctions and the basement membrane of endothelial cells, which leads to BBB damage, increased permeability and vasogenic brain edema. Activation of MMP-12 induces myelin basic protein (MBP) degradation^15^. Other MMP-12 substrates include pro-TNFα, α1-antitrypsin, tissue factor pathway inhibitor, plasminogen, and N-cadherin^15^,^16^,^17^,^18^,^19^. MMP-12 expression is upregulated after intracerebral hemorrhage, which accounts for approximately 15–20% of all strokes. After intracerebral hemorrhage, MMP-12 knockout mice exhibited improved sensorimotor function compared to their wild-types^20^. Although the MMP-12 upregulation after neonatal hypoxic-ischemic brain injury has recently been demonstrated^21^, few attempts have been made to study its role in the ischemic stroke and the impact of its knockdown on brain damage.

Our preliminary studies showed an increased mRNA expression of MMP-12 for seven days after ischemia and reperfusion. Of all the MMPs studied, MMP-12 exhibited the highest upregulation. Based on the reported data and our preliminary studies, we hypothesize that specific knockdown of MMP-12 by shRNA-mediated gene silencing after focal cerebral ischemia would reduce brain damage and inhibit cell death. We investigated the effect of intravenous administration of MMP-12 shRNA expressing plasmid on ischemic brain damage and apoptotic cell death in a rat model after middle cerebral artery occlusion followed by reperfusion. This is the first study to explore the deleterious role of MMP-12 after ischemic stroke.

## Results

### MMP-12 is upregulated after focal cerebral ischemia

Sham group animals that are operated as per the described MCAO procedure in the methods section except for the insertion of the monofilament served as controls. MCAO-subjected animals treated with a plasmid containing a vector that is inserted with a scrambled shRNA sequence served as vehicle controls. The data obtained from the animals that died during the course of the study and those that did not demonstrate neurological signs after MCAO procedure are not considered for evaluation.

**Figure 1:**
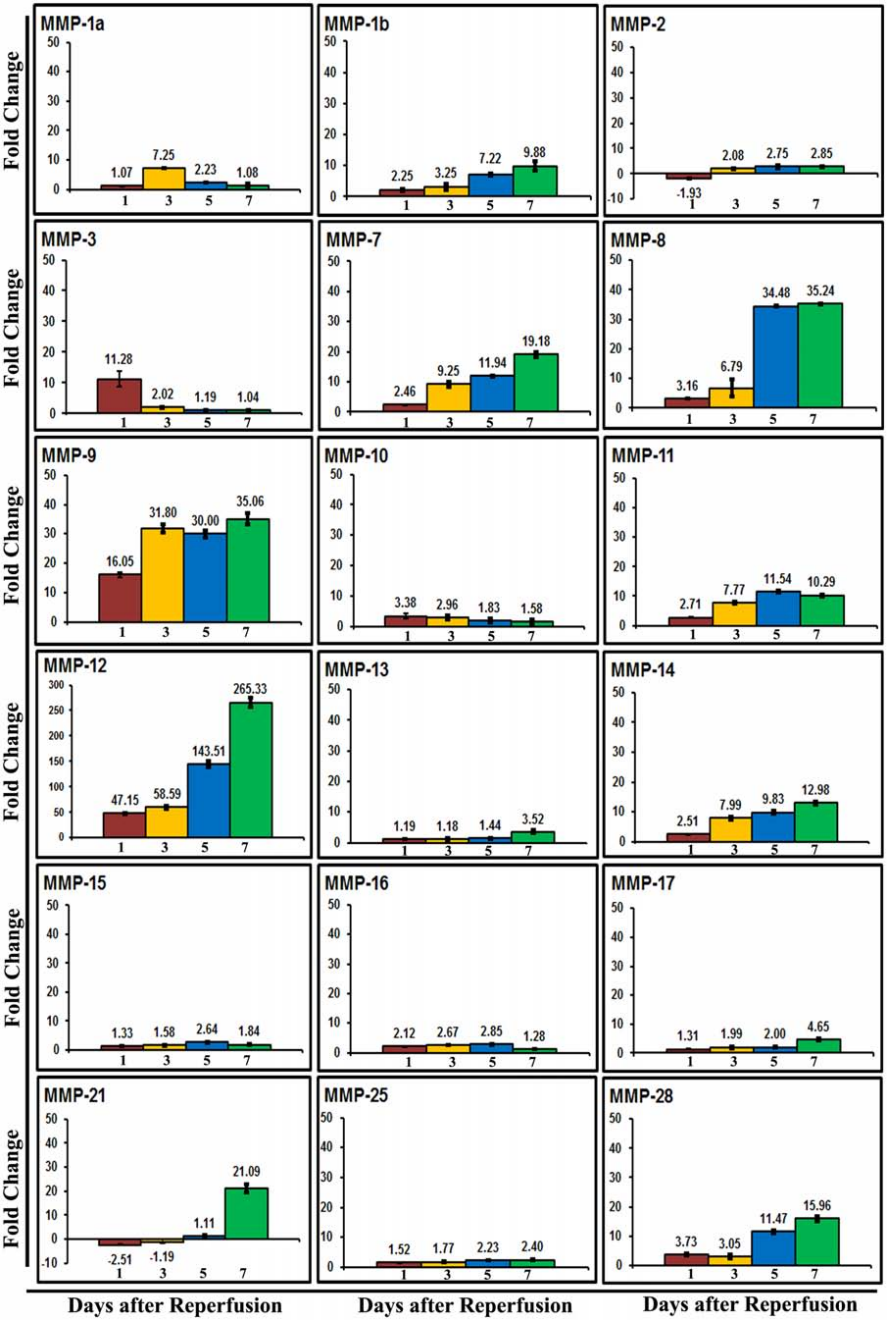
Regulation of MMPs in rats after focal cerebral ischemia. Quantitative real-time PCR data of MMPs in the ischemic brains of rats subjected to a two-hour MCAO procedure followed by various reperfusion times. Error bars indicate SD. n = 6. Values on y-axis represent fold change compared to sham controls.

**Figure 2:**
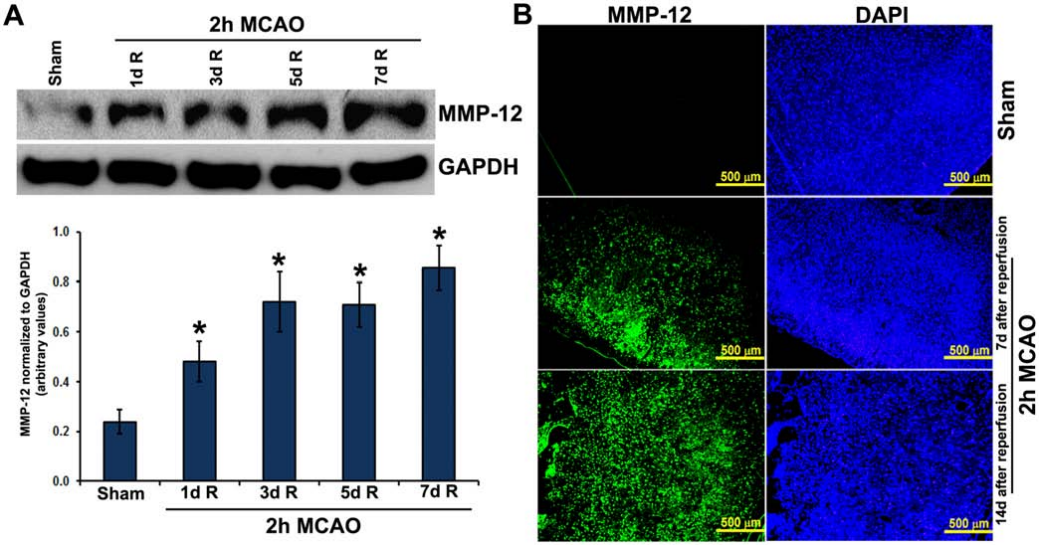
MMP-12 protein expression in rats subjected to focal cerebral ischemia and reperfusion. (A) Immunoblot analysis depicting the protein expression of MMP-12 in the ipsilateral brain regions of rats subjected to ischemia and reperfusion. GAPDH was used as a loading control. Bar graph represents the quantification of MMP-12 protein expression. n = 6. R = reperfusion. *p < 0.05 vs. sham. (B) Immunofluorescence analysis depicting MMP-12 protein expression (green fluorescence) in the ipsilateral brain regions of sham controls and the rats subjected to a two-hour MCAO procedure followed by seven days or fourteen days reperfusion. Nuclei were stained with DAPI. n = 6.

**Figure 3:**
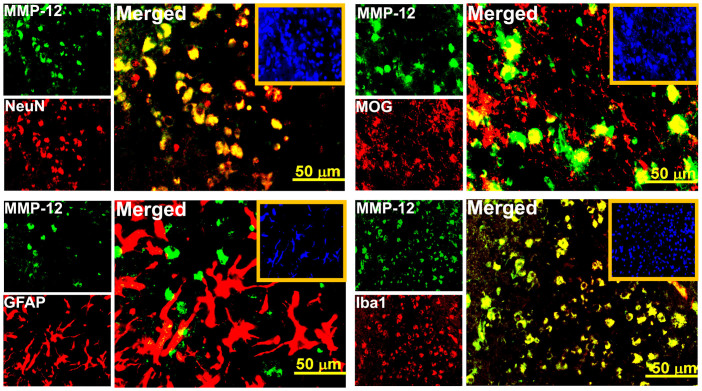
Cellular localization of MMP-12 in neuronal cells of rats, which were subjected to focal cerebral ischemia and reperfusion. Immunofluorescence analysis showing the protein expressions of MMP-12 (green fluorescence) and NeuN/GFAP/MOG/Iba1 (red fluorescence) in the ipsilateral brain regions of rats subjected a two-hour MCAO procedure followed by seven days reperfusion. Yellow fluorescence in the merged images indicates the cellular localization of MMP-12. Nuclei were stained with DAPI. Insets in the merged images show DAPI staining. n = 6.

The fold changes of mRNA for each MMP family member relative to their sham control values were quantified in the ipsilateral brain tissues of rats at 1, 3, 5 and 7 days after reperfusion subsequent to a two-hour right MCAO. The expression profile of several rat MMPs in terms of fold changes compared to controls are shown in Figure 1. The changes in the expression of MMP-2, -10, -13, -15, -16, -17, and -25 were not prominent. Certain MMPs such as MMP-1b, -7, -8, -9, -11, -12, -14, and -28 increased day 1 through day 7 post-MCAO. MMP-3 showed a prominent increase in its mRNA expression on day 1 post-MCAO, but its levels were back to normal at the subsequent time points. In contrast, prominent upregulation of MMP-21 was noticed only on day 7 post-MCAO. Of all the MMPs studied, MMP-12 exhibited most marked upregulation at all time points tested during the reperfusion period. The increase was gradual from day 1 through day 7 post-MCAO (Figure 1). MMP-12 mRNA levels were approximately 47 and 265 folds higher than the controls on days 1 and 7 post-MCAO procedure, respectively. MMP-12 protein expression was significantly (p < 0.05) increased at all-time points after reperfusion (Figure 2A). MMP-12 protein expression was prominent for 14 days following two hours of ischemia and reperfusion (Figure 2B). The expressed MMP-12 in the ischemic rat brains was localized to neurons, oligodendrocytes and microglia, but not astrocytes (Figure 3).

### MMP-12 knockdown attenuates ischemic brain damage

**Figure 4:**
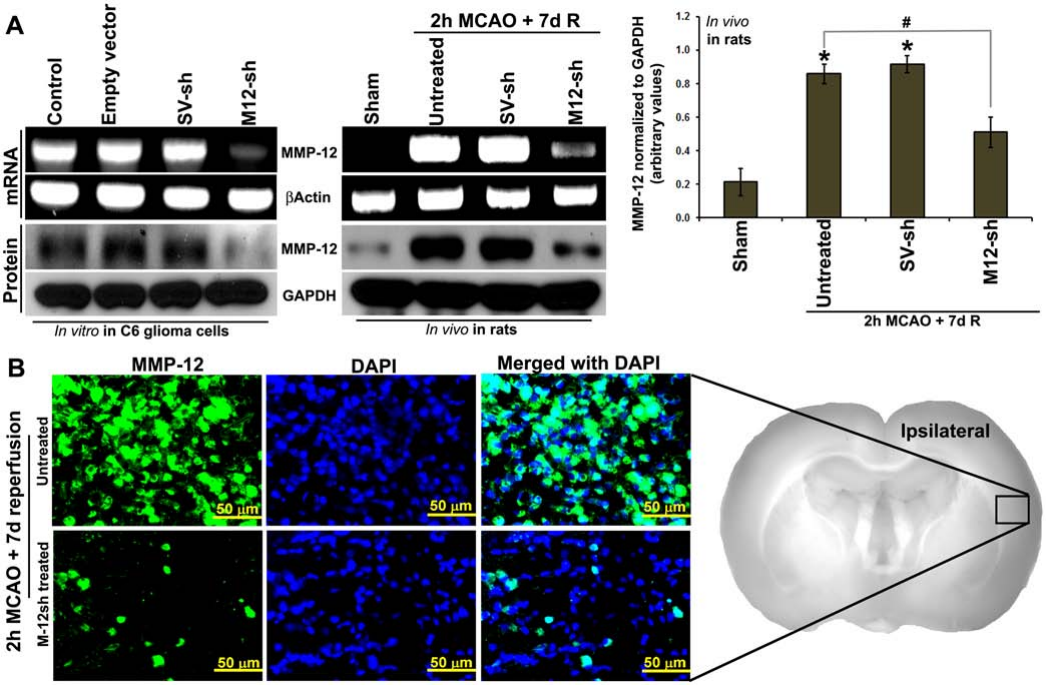
*In vitro and in vivo* efficiency of plasmids expressing MMP-12 shRNA. (A) RT-PCR and immunoblot analysis depicting the mRNA and protein expression, respectively, of MMP-12 after transfection of C6 rat glioma cell lines with plasmids containing an empty vector, a vector ligated with a scrambled sequence (SV-sh) and a vector ligated with MMP-12 shRNA (M-12-sh). βActin and GAPDH were used as loading controls. n = 3. Expression of MMP-12 in the ischemic brain regions of rats subjected a two-hour MCAO followed by seven days reperfusion and/or treated with SV-sh or M-12-sh. Bar graph represents the quantification of MMP-12 protein expression. n = 6. *p < 0.05 vs. sham; ^#^p < 0.05 vs. untreated. (B) Immunofluorescence analysis depicting the protein expressions of MMP-12 (green fluorescence) in untreated and M-12-sh treated rats subjected to a two-hour MCAO procedure followed by seven days reperfusion. Nuclei were stained with DAPI. n = 6.

**Figure 5:**
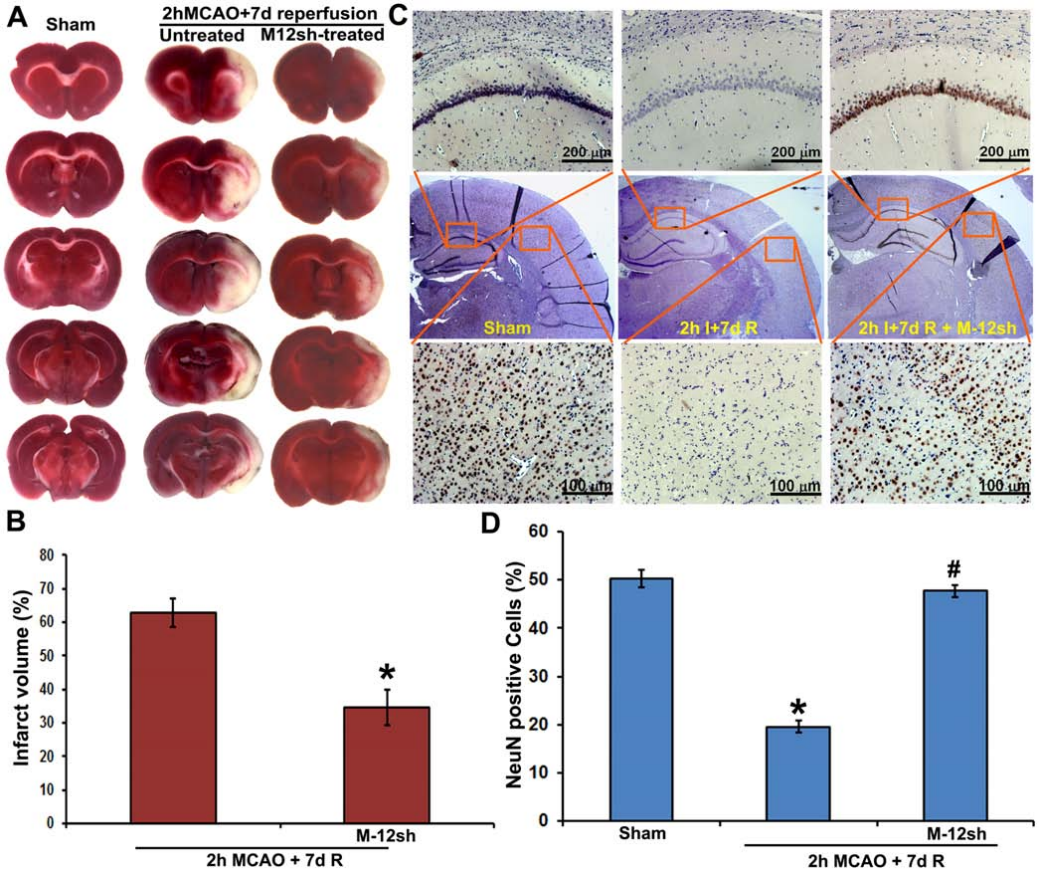
*In vivo* efficacy of plasmids expressing MMP-12 shRNA. (A) Representative TTC staining images of the rat coronal brain sections of sham-operated, untreated MCAO-subjected and MMP-12 shRNA (M-12sh) treated MCAO-subjected rats sacrificed seven days after reperfusion. n = 6. The white-colored areas represent the infarct regions in these sections, and the red-colored areas represent normal areas. (B) Quantification of infarct volume from TTC stained sections using image analysis software. The possible influence of edema on infarct volume was corrected by standard methods (volume of contralateral hemisphere − volume of non-ischemic ipsilateral hemisphere), with infarcted volume expressed as a percentage of the contralateral hemisphere. n = 6. Values are expressed as mean ± SEM; *p < 0.05 vs. untreated, MCAO-subjected animals. (C) Immunohistochemical analysis of ipsilateral rat coronal brain sections depicting DAB staining (brown), representative of neurons. The missing NeuN-DAB staining in injured brain sections due to neuronal loss is restored after MMP-12 knockdown. Brain sections are counterstained with hematoxylin for nuclear localization. Results are from six independent sections obtained from six different rats. I-ischemia; R-reperfusion. (D) Quantification of NeN positive cells in the cortex of ipsilateral brain. n = 6. *p < 0.05 vs. sham; ^#^p < 0.05 vs. untreated MCAO-subjected animals.

Plasmids expressing MMP-12 shRNA were constructed in pSilencer™ 4.1-CMV neo vector and synthesized from the overnight bacterial culture. Sequences of the shRNA were confirmed. In addition, the reduced mRNA and protein expressions of MMP-12 in C6 rat glioma cells transfected with plasmids expressing MMP-12 shRNA have validated the *in vitro* efficiency of the designed, constructed, and synthesized MMP-12 shRNA expressing plasmids (Figure 4A). Similarly, nanoparticle-mediated intravenous delivery of MMP-12 shRNA expressing plasmids reduced the mRNA and protein expression of MMP-12 in the ischemic brain regions, which in turn demonstrated the *in vivo* efficiency of MMP-12 shRNA expressing plasmids (Figure 4A). As expected, similar administration of a plasmid expressing vector inserted with a scrambled sequence, serving as a vehicle control, did not reduce MMP-12 expression. Unchanged expression profile of either βActin or GAPDH across various treatment groups confirmed loading consistency for the gels. The MMP-12 shRNA plasmids also decreased MMP-12 protein expression in histologic sections stained by immunofluorescence (Figure 4B). After we noticed the *in vivo* efficiency of these plasmids expressing MMP-12 shRNA, we administered them as nanoparticles formulated with an *in vivo* transfection reagent to another set of rats to evaluate the extent of brain damage by performing 2,3,5-triphenyltetrazolium chloride (TTC) staining. TTC staining revealed a massive infarct size in the cortex and striatal regions of ipsilateral hemisphere in rats subjected to a two hour MCAO procedure followed by seven days of reperfusion (Figure 5A). As expected, we did not notice any infarction in the respective brain sections of sham-operated rats or in the contralateral sides of the operated animals. Percent infarct size was significantly (p < 0.05) reduced from 62.87 ± 4.13 (mean ± SEM) to 34.67 ± 5.39 after the intravenous nanoparticle-mediated delivery of MMP-12 shRNA expressing plasmid in rats subjected to ischemia and reperfusion (Figure 5B). In addition, DAB-immunostaining performed for NeuN on the ipsilateral coronal brain sections obtained from various groups of rats indicated a remarkable loss of neurons in the ischemic brain regions of animals subjected to ischemia and reperfusion (Figure 5C). Percentage of NeuN positive cells in the cortex of ipsilateral brain sections were significantly (p < 0.001) reduced after ischemia and reperfusion (Figure 5D). However, treatment with plasmids expressing MMP-12 shRNA one-day after reperfusion significantly (p < 0.001) reversed the neuronal loss in the infarcted brain (Figure 5C and 5D).

### MMP-12 knockdown alters MMP-9, myelin basic protein and apoptosis

**Figure 6:**
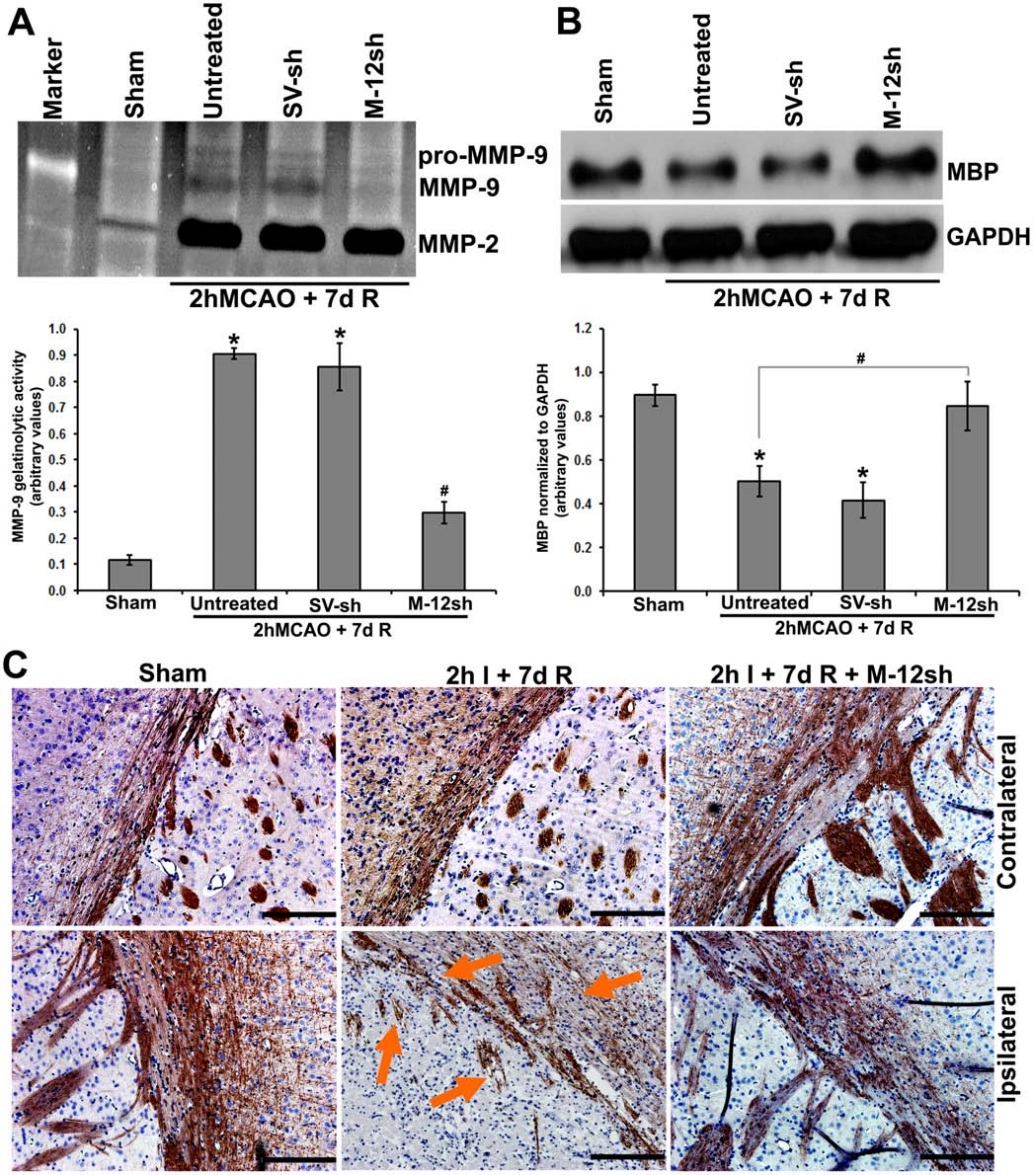
Effect of MMP-12 knockdown on other possible MMP-12 substrates. (A) Gelatin zymogram showing MMP levels in sham control and ischemic brains of rats subjected to a two-hour MCAO followed by seven days reperfusion without treatment and treatment with plasmids containing a vector ligated with a scrambled sequence (SV-sh) and a vector ligated with MMP-12 shRNA (M-12-sh). Quantification of MMP-9 gelatinolytic activity. n = 6. *p < 0.05 vs. sham; ^#^p < 0.05 vs. untreated. R = reperfusion (B) Immunoblot showing the protein expression of MBP in the ischemic brains of rats subjected to a two-hour MCAO followed by seven days reperfusion with and without treatments. GAPDH was used as a loading control. Quantification of MBP protein expression. n = 6. *p < 0.05 vs. sham; ^#^p < 0.05 vs. untreated. (C) DAB immuno-staining depicting the protein expression of MBP in the contralateral and ipsilateral rat coronal brain sections obtained from various groups of rats. Arrows demonstrate the marked loss of MBP-immunostained axonal processes with clear structural abnormalities of rarefaction and fragmentation. Brain sections are counterstained with hematoxylin for nuclear localization. n = 6. Scale bar = 200 μm.

Gelatin zymography revealed prominent increases in MMP-2 and pro- and active-MMP-9 expression in rats subjected to a two hour MCAO followed by seven days reperfusion (Figure 6A). MMP-12 knockdown one day post-MCAO significantly (p < 0.001) reduced the increased MMP-9 levels but did not affect MMP-2. Myelin basic protein (MBP) protein expression was significantly (p < 0.05) downregulated, as expected, in rats that were subjected to focal cerebral ischemia and reperfusion (Figure 6B). Increased MBP protein expression after MMP-12 knockdown in ischemia-subjected rats demonstrated that the silencing of MMP-12 inhibits MBP degradation and reverts MBP levels back to near normal (Figure 6B). Immunohistochemical analysis of the brain sections obtained from rats subjected to ischemia and reperfusion showed a marked loss of MBP-immunostained axonal processes with clear structural abnormalities of rarefaction and fragmentation in the ipsilateral brain regions compared to their contralateral counterparts (Figure 6C). MMP-12 knockdown one day after reperfusion inhibited myelin degradation in the ischemic tissue and showed dense myelin fibers, which are comparable to those present in the respective contralateral brain regions.

**Figure 7:**
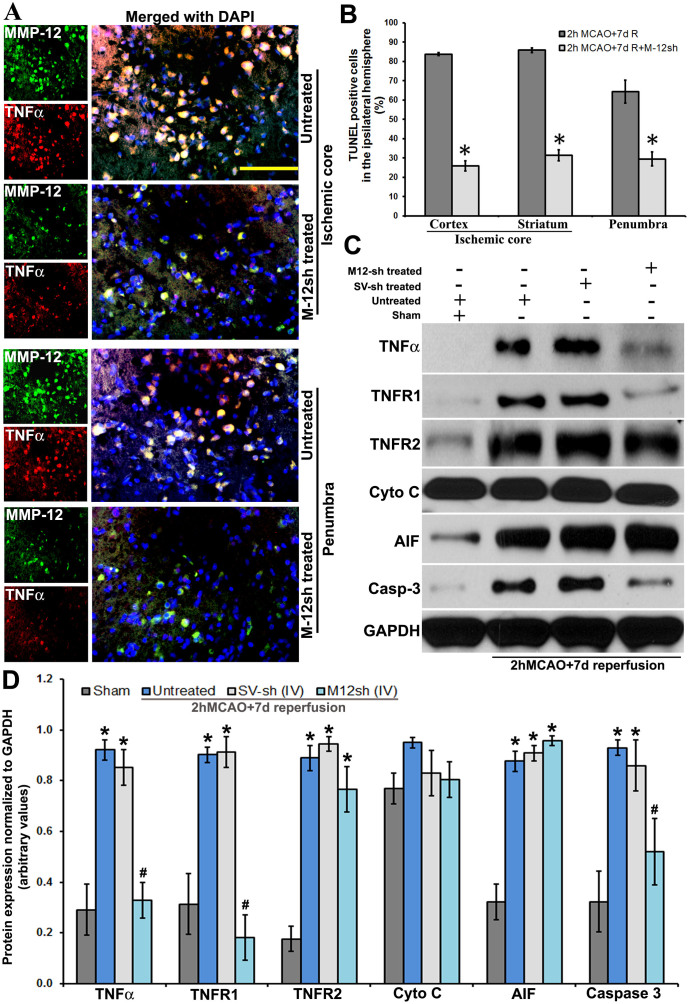
Effect of MMP-12 knockdown after focal cerebral ischemia on TNFα-mediated apoptosis. (A) Immunofluorescence analysis showing the protein expressions of MMP-12 (green fluorescence) and TNFα (red fluorescence) in the ischemic core and the penumbra of rats subjected a two-hour MCAO procedure followed by seven days reperfusion with and without treatments. Nuclei were stained with DAPI (blue fluorescence). Yellow fluorescence in the merged images indicates the colocalization of MMP-12 and TNFα. n = 6. Scale bar = 100 μm. (B) TUNEL assay on the paraffin-embedded coronal brain sections of rats subjected to a two-hour MCAO procedure followed by seven days reperfusion with and without treatments. TUNEL-positive cells were quantified in the ipsilateral regions of rat brain that consisted of the ischemic core and the penumbra. n = 6. Values are expressed as mean ± SEM. *p < 0.05 compared to untreated animals subjected to a two-hour MCAO followed by seven days reperfusion. (C) Immunoblots showing the protein expression of various apoptotic molecules in the ischemic brains of rats subjected to a two-hour MCAO followed by seven days reperfusion with and without treatments. SV-sh: treated with plasmid expressing a scrambled sequence (vehicle control). M-12sh: treated with plasmid expressing MMP-12 shRNA. n = 6. GAPDH was used as a loading control. (D) Quantification of immunoblots. n = 6. *p < 0.05 vs. sham; ^#^p < 0.05 vs. untreated.

Earlier reports indicated pro-TNFα as one of the MMP-12 substrates^15^. Our results show that after MMP-12 knockdown, TNFα expression was reduced in both the ischemic core and the penumbra (Figure 7A). Colocalization of MMP-12 with TNFα in the ischemic brain regions was missing after treatment with plasmids expressing MMP-12 shRNA (Figure 7A). Further, percent terminal deoxy nucleotidyl transferase-mediated nick end labeled (TUNEL) cells in the ipsilateral hemisphere were significantly (p < 0.05) reduced from 83.77 ± 2.47 (cortex region of the ischemic core), 85.84 ± 2.13 (striatum region of the ischemic core), and 64.29 ± 11.05 (penumbra) to 25.93 ± 1.30, 31.4 ± 3.06, and 29.54 ± 1.86, respectively, in rats after MMP-12 knockdown compared to untreated MCAO subjected rats (Figure 7B). The reduction in TUNEL positive cells was noted in both the ischemic core and the penumbra. As expected, TNFα-mediated apoptotic pathway molecules such as TNFR1 and caspase-3 protein expression were significantly (p < 0.05) reduced in the ischemic brains of rats that were subjected to MMP-12 knockdown (Figures 7C and 7D). However, MMP-12 knockdown did not affect the apoptotic proteins associated with the mitochondrial pathway such as cytochrome C and apoptosis-inducing factor (AIF), which were tested in the present study (Figures 7C and 7D).

## Discussion

The role of MMP-12 is well documented in lung diseases such as COPD, emphysema, and asthma. It is still perplexing why stroke researchers have paid little attention to it despite its possible pathological role in the context of ischemic stroke. The results of this study indicate a pathological role for MMP-12 in ischemic stroke and highlight a potential therapy to mitigate the damage after ischemic stroke by reducing MMP-12 expression.

Induction, expression, and release of MMPs after cerebral ischemia and/or reperfusion take place at all times during the ischemic process. MMPs likely play a detrimental role in brain ischemia and reperfusion by degrading extracellular matrix and the BBB, which can lead to vasogenic brain edema and secondary brain injury. Pre-clinical studies that utilized MMP inhibitors, MMP neutralizing antibodies, MMP knockout models, and siRNAs/shRNAs to specific MMPs reduced vasogenic brain edema and secondary brain injury and have shown neuroprotective effects^8^,^22^,^23^,^24^,^25^,^26^,^27^,^28^. Of all the MMPs studied and reported to date, considerable emphasis was given to gelatinases, MMP-2, and MMP-9. In this study, we noticed a remarkable upregulation of MMP-12 subsequent to focal cerebral ischemia in rats. Of all the MMPs we studied, MMP-12 mRNA showed the highest upregulation with a steady increase over time after reperfusion. Although recent reports indicated a marked elevation of MMP-12 RNA transcript in rat and mouse models of intracerebral haemorrhage, its regulation after cerebral ischemia has neither been studied nor reported to date^20^,^29^. To our knowledge, there is only one study that demonstrated the upregulation of MMP-12 after hypoxic-ischemic brain injury in mice^21^. The authors reported increased mRNA and protein expression of MMP-12 in the ipsilateral hemisphere, which are in agreement with our results. Further, the elevated MMP-12 levels after either ischemia or intracerebral hemorrhage were correlated with the extent of brain tissue loss and secondary injury^20^,^21^.

MMP-12 expression has been identified in all neuronal cells of ischemic rat brain except astrocytes, which is in agreement with the earlier reports^21^. In contrast, MMP-12 expression in astrocytes has been reported after intracerebral hemorrhage^30^. Besides elastase activity, MMP-12 shows broad substrate specificity on ECM proteins such as fibronectin, laminin, vitronectin, type IV collagen, and heparin sulfate^15^. Accordingly, MMP-12 not only digests elastin but also degrades the basement membrane, which allows macrophages to penetrate into injured tissue during inflammation^31^. Recently, it was reported that the upregulated cerebral MMP-12 during aging enhances neuroinflammation by facilitating recruitment of bone marrow-derived microglia into the brain^32^. Type I gelatin and myelin basic protein have also been identified as MMP-12 substrates^15^,^33^. Several other MMP-12 substrates include α1-antitrypsin, tissue factor pathway inhibitor, plasminogen, and N-cadherin^15^,^16^,^17^,^18^,^19^. MMP-12 cleaved alpha 1-antitrypsin and released TNF from a pro-TNF fusion protein^15^. In addition to impaired TNFα release in MMP-12 knockout mice, MMP-12 is involved in the processing of pro-TNFα to active TNFα^15^,^34^. TNFα is well known for its role in inflammation and apoptosis. These reports on MMP-12 support the possibility of its role in the pathogenesis of ischemic stroke. Our results further strengthen this observation and demonstrate the importance of silencing MMP-12 by administering MMP-12 shRNA expressing plasmids in order to achieve a significant neuronal protection after ischemic stroke (Figure 5). Although several controversies exist regarding the *in vivo* administration of shRNA expressing plasmids, we were successful in delivering MMP-12shRNA expressing plasmids as nanoparticles to ischemic brain cells. Our study is the first to demonstrate the non-viral, nanoparticle-mediated *in vivo* delivery of shRNA expressing plasmids to brain in the context of ischemic stroke. Despite the possible toxicity associated with the intravenous administration of these shRNA expressing plasmids, we were successful in demonstrating the *in vivo* efficiency and efficacy of these plasmids. Our future studies will explore the delivery of these plasmids to brain that would avoid the possible toxicities associated with widespread expression changes.

MMP-12 upregulated in the brain during brain development is vital for myelination^35^,^36^. In contrast, in the ischemic rat brains, structural abnormalities of rarefaction and fragmentation of myelin, including reduced MBP expression were identified. These results are in agreement with other reports indicating MMP-12 positive cells were identified in patients with multiple sclerosis and chronic demyelinating Theiler murine encephalomyelitis^37^,^38^. In this study, MMP-12 suppression corrected the myelin-associated abnormalities that occurred after cerebral ischemia and reperfusion. In addition, MMP-12 knockdown inhibited the increased MMP-9 activity in ischemic brains. These results supported the earlier reports indicating the possible role of MMP-12 in the induction of other MMPs, such as MMP-9^39^. A pathological role of MMP-9 after cerebral ischemia and reperfusion is well-known and has been addressed in the beginning of this section. Based on these findings, we believe that the inhibition of myelin degradation and MMP-9 activation could be the possible mechanisms of neuronal protection associated with MMP-12 knockdown after focal cerebral ischemia. In addition, as stated earlier, MMP-12 silencing inhibited TNFα-mediated apoptotic cell death which could be due to its control over TNFα (Figure 7). Inhibition of apoptosis in the ipsilateral brain regions could be one of the major mechanisms of protection after MMP-12 knockdown subsequent to cerebral ischemia and reperfusion.

This study clearly demonstrates the pathological role of MMP-12 after focal cerebral ischemia and reperfusion as well as highlights the possible mechanisms of protection after MMP-12 knockdown. Although this study has some limitations such as lack of experimentation that demonstrate the type of TUNEL positive cells, paucity of sufficient data on toxicity upon intravenous delivery of MMP-12 shRNA expressing plasmids which can lead to widespread expression changes and lack of clarity on the time point following ischemia at which MMP-12 activity is actually detrimental/beneficial, MMP-12 is regarded as a promising therapeutic target for ischemic stroke. In the present study, we did not notice any difference in the percent mortality in untreated and treated rats which were subjected to MCAO procedure. Our future studies will address the above-mentioned limitations and explore the effective dose, route, and time of administration of MMP-12 shRNA expressing plasmids, which could offer significant neuronal protection after cerebral ischemia and reduce brain damage with improved long-term neurological recovery.

## Methods

### Ethical statement

All experiments were performed to comply with the ARRIVE (Animal Research: Reporting *In Vivo* Experiments) guidelines. The Institutional Animal Care and Use Committee (IACUC) of the University of Illinois College of Medicine at Peoria approved all surgical interventions and post-operative animal care. All animal experiments were conducted in accordance with the IACUC guidelines. In addition, all the procedures that were performed on the animals were in compliance with the approved IACUC protocol.

### Antibodies

Anti-MMP-12, anti-MMP-9, anti-GFAP, anti-MOG, anti-Iba1, anti-TNFα, anti-TNFR1, anti-TNFR2, anti-caspase-3, anti-cytochrome C, anti-AIF, and anti-MBP antibodies were obtained from Santa Cruz Biotechnology (Santa Cruz, CA). Anti-NeuN antibody was obtained from Millipore (Billerica, MA). Anti-glial fibrillary acidic protein (GFAP) was obtained from Dakocytomation (Carpinteria, CA). Glyceraldehyde-3-phosphate dehydrogenase (GAPDH) antibody was obtained from Novus Biologicals (Littleton, CO).

### Cell culture and transfection conditions

C6 rat glioma cells obtained from ATCC were grown in ATCC-formulated F-12K medium supplemented with 2.5% fetal bovine serum (Hyclone, Logan, UT), 15% horse serum, and 1% penicillin/streptomycin (Invitrogen, Carlsbad, CA) at 37°C in a humidified atmosphere containing 5% CO_2_. C6 glioma cells cultured in 6-well and 100 mm plates were transfected with empty vector (pSilencer™ 4.1-CMV neo), vector ligated with a scrambled sequence (SV-sh), or M-12sh plasmid by using an *in vitro* transfection reagent, Fugene® HD (Roche Diagnostics, Indianapolis, IN) according to the manufacturer's instructions.

### Design, construction and synthesis of shRNA expressing plasmids

We designed and synthesized rat MMP-12 shRNA (M-12sh) plasmid to silence the gene expression of MMP-12. We used pSilencer™ 4.1-CMV neo vector obtained from Ambion (Austin, TX) to construct M-12sh along with shRNA for a scrambled sequence which served as a vehicle control. Rat MMP-12 target sequence (5′CTGCTGCATTTCCTGGAGTT3′) was chosen according to Ambion's siRNA design online tool. This target sequence was used to design the below invert repeat MMP-12 shRNA sequences.

Forward primer: 5′GATCCACTCCAGGAAATGCAGCAGTTCAAGAGACTGCTGCATTTC

CTGGAGTTTA3′

Reverse primer: 5′AGCTTAAACTCCAGGAAATGCAGCAGTCTCTTGAACTGCTGCATTT

CCTGGAGTG3′

Invert repeat sequences synthesized for rat MMP-12 were laterally symmetrical, causing them to be self-complimentary with a 9 bp mismatch in the loop region. Oligonucleotides/ultramers were annealed, and the annealed product was ligated to the vector at the BamHI and HindIII sites according to the manufacturer's instructions. The resultant vectors were transformed into chemically competent *E. coli* cells (JM109 competent cells) and cultured overnight. MMP-12 shRNA expressing plasmid was synthesized from the overnight bacterial culture by using QIAGEN plasmid mini/maxi kit according to the manufacturer's protocol. Positive clones were confirmed by gene sequencing analysis.

### Animals and experimental design

To exclude any gender-related differences in the severity of the brain injury, only male Sprague–Dawley rats were used in this study. A total of 80 rats weighing 200–240 g were procured from Harlan Laboratories and maintained in a specific pathogen free Laboratory Animal Care Facility of University of Illinois College of Medicine at Peoria. Animals were housed in a 12-h light/dark cycle at a controlled temperature and humidity with free access to food and water. After the animals reached a weight of 260 ± 5 g, they were assigned to four groups, consisting of at least 20 animals. Based on the literature and after considering a 10–20% mortality associated with middle cerebral artery occlusion (MCAO) procedure, we needed this number of animals per each group to attain a proper statistical analysis. Animals in group I were used as sham controls. Remaining animals were subjected to the MCAO procedure. Animals that did not show post-stroke symptoms after MCAO procedure were excluded from the study. Animals that showed post-stroke symptoms after MCAO procedure were randomly allocated to groups 2, 3, and 4. Group 2 animals were subjected to the MCAO procedure with no further treatments. Group 3 and Group 4 animals were intravenously injected with SV-sh and M-12sh formulations, respectively, at a dose of 1 mg/kg body weight via tail vein 24 hours post-MCAO procedure. All animals from the four groups were sacrificed seven days post-MCAO procedure. The brain tissues obtained from these animals were utilized for various experimental procedures. All the slides processed for immunohistochemical analysis were evaluated by a pathologist blinded to the treatments.

### Experimental MCAO model

After the animals reached a weight of 260 ± 5 g, they were subjected to right MCAO procedure as previously described^40^. Briefly, a ventral midline incision (~25 mm) was made in the neck and the right common carotid, internal carotid, and external carotid arteries were surgically exposed. The external carotid artery was permanently ligated rostral with one ligature. A microaneurysm clip was applied to the external carotid artery near its bifurcation with the internal carotid artery. A small puncture opening was made in the external carotid artery. The monofilament was inserted through the opening, and the other loose ligature was tightened around the lumen containing the monofilament. The microaneurysm clip was removed from the external carotid artery, and the monofilament was then gently advanced from the lumen of the external carotid artery into the internal carotid artery for a distance of ~19 to 20 mm beyond the bifurcation of the common carotid artery. The skin on the neck incision was closed with surgical wound clips. To restore the blood flow 2 hours after MCA occlusion, the surgical site was re-opened by removing the wound clips. The microaneurysm clip was removed, the knot was loosened, the monofilament was withdrawn, and the knot was re-tied to stop bleeding. The skin was sutured to close the neck incision. After MCAO procedure, all animals were treated with appropriate analgesics and antibiotics and subjected to the approved post-procedural care.

### Formulation preparation for *in vivo* administration

MMP-12 shRNA expressing plasmids were formulated as nanoparticles, which are sufficiently small to diffuse into the tissues and enter the cells by endocytosis. SV-sh and M-12sh expressing plasmids, synthesized by using QIAGEN plasmid maxi kit, were further purified by phenol-chloroform extraction procedure. *In vivo*-JetPEI, obtained from Polyplus transfection (Illkirch, France) contains a cationic polymer that efficiently condenses plasmid DNA. Plasmids were quantified and formulated with *in vivo*-JetPEI and sterile water for injection using aseptic conditions according to the manufacturer's instructions.

### RNA extraction and cDNA synthesis

Total RNA was extracted using TRIzol reagent (Invitrogen, Carlsbad, CA) from (1) untreated and hypoxia-induced cortical neurons, (2) untreated and transfected C6 glioma cells, (3) sham controls, (4) ipsilateral hemisphere of rats subjected to various ischemia and reperfusion conditions, and (5) ipsilateral hemisphere of those animals subjected to SV-sh and M-12sh treatments. Briefly, total RNA was extracted with TRIzol, precipitated with isopropyl alcohol, washed in ethanol, and resuspended in RNase-free water. 1 μg of total RNA was reverse transcribed into first-strand cDNA using the Transcriptor First Strand cDNA Synthesis Kit (Roche, Indianapolis, IN) according to the manufacturer's instructions. The cDNAs obtained were used for reverse transcription polymerase chain reaction (RT-PCR) analysis and real-time PCR analysis.

### Quantitative real-time PCR

The forward and reverse primer sequences of rat MMPs used in this study has been reported earlier by our group^41^. Reaction set-up for each cDNA sample was assembled using the IQ™ SYBR® Green Supermix kit (Bio-Rad Laboratories, CA) as per the manufacturer's instructions. Samples were subjected to forty cycles at 95°C for fifteen seconds and 60°C for one min in iCycler IQ (Multi Color Real-Time PCR Detection System, Bio-Rad Laboratories, CA). Data were collected and recorded using the iCycler IQ software (Bio-Rad Laboratories, CA) and expressed as a function of the threshold cycle (Ct), which represents the number of cycles at which the fluorescent intensity of the Sybr Green dye is significantly above than that of the background fluorescence. The housekeeping gene, β-actin was used for normalization of MMPs expression. Average Ct values were normalized with average Ct values of β-actin. After normalization of the Ct values, fold differences were calculated by using the formula 2^^^-(ΔCt of Test)/2^^^-(ΔCt of controls).

### RT-PCR analysis and agarose gel electrophoresis

RT-PCR analysis was performed using cDNAs obtained from the brains of various treatment groups using GoTaq® Green Master Mix (Promega) as per the manufacturer's instructions. RT-PCR was set up in C1000 Touch Thermo cycler (Bio-Rad Laboratories, CA) using the following PCR cycle: [95°C for 5 min, (95°C for 30 sec, 58–62°C for 30 sec, and 72°C for 30 sec) x 40 cycles, and 72°C for 10 min]. RT-PCR end product was resolved on a 1.6% agarose gel, visualized, and photographed under UV light. The housekeeping gene β-actin was used to verify that similar amounts of cDNA were loaded in all the lanes.

### Immunoblot analysis

In order to study the expression of various proteins in tissue lysates of untreated and treated ipsilateral brain hemispheres of MCAO subjected rats as well as the respective brain tissue of sham controls, immunoblot analysis was performed as previously described^40^. Briefly, ipsilateral brain hemispheres of rats from various groups were suspended in 0.2 mL of homogenization buffer, homogenized using a Tissue Tearor (Biospec Products, Inc.), and followed by sonication. Tissue homogenate was centrifuged at 15,000 × *g* for 30 min at 4°C, and the protein levels in the supernatant were determined using the BCA assay (Pierce, Rockford, IL). Samples [equal amount (30–50 μg) of total protein/well] were subjected to 12–14% SDS-PAGE based on the specifications of the protein, and the protein bands on the gel were transferred onto nitrocellulose membranes. The membranes were processed with primary antibodies followed by appropriate HRP-conjugated secondary antibodies. Immunoreactive bands were visualized using chemiluminescence ECL Western blotting detection reagents on Hyperfilm-MP autoradiography film (Amersham, Piscataway, NJ). Immunoblots were re-probed and processed with GAPDH antibody and the measurements were normalized to loading controls.

### Brain tissue fixation and sectioning

On the seventh day post-MCAO procedure, animals from all four groups were placed under deep anesthesia with pentobarbital and perfused through the left ventricle with 70–100 mL of phosphate buffered saline (PBS), followed by 100–150 mL of 10% buffered formalin (Fisher Scientific, NJ). The brains of the animals from various treatment groups were then removed, fixed in 10% buffered formalin, and embedded in paraffin. Serial coronal brain sections were cut at a thickness of 5–6 μm with a microtome.

### Immunohistochemistry

Paraffin-embedded brain sections of various groups of animals were subjected to immunohistochemical analysis. Briefly, paraffin-embedded rat coronal brain sections were de-paraffinized, subjected to antigen retrieval, permeabilized, and processed with MMP-12, NeuN or MBP primary antibodies. The sections were then washed in PBS and incubated with the appropriate HRP-conjugated secondary antibodies for one hour at room temperature. After one hour, the sections were washed in PBS and incubated with diamino benzidine (DAB) for 30 min. The slides were further washed with sterile water, stained with hematoxylin, and dehydrated. The slides were then covered with glass cover slips, and photomicrographs were obtained. Immunofluorescence analysis was performed to identify, (1) the changes in the expression of MMP-12 protein after MCAO procedure, (2) the colocalization of MMP-12 protein with neuronal cells, and (3) the colocalization of MMP-12 protein with TNFα. In case of immunofluorescence analysis, brain sections subjected to primary antibodies treatment were processed by appropriate fluorescent-labeled secondary antibodies, counterstained with DAPI, cover slipped, and observed using a confocal microscope (Olympus Fluoview). Negative controls (without primary antibody or using isotype specific IgG) were maintained for all the samples.

### TTC staining and measurement of infarct size

Animals from various groups allocated for 2,3,5-triphenyltetrazolium chloride (TTC; Sigma) staining procedure were deeply anesthetized with pentobarbital and then decapitated. Brains were then removed rapidly and subjected to TTC staining and infarct size measurement. Briefly, rat brains were placed in pre-chilled adult rat brain matrix (Kent Scientific Corporation). Matrix containing the brain tissue was then placed in a freezer at −70°C for 5–8 min and sliced into 2-mm-thick coronal sections. These slices were stained in 2% TTC for 30 min at 37°C in the dark. The infarction area and hemisphere area of each section was traced and measured using Image J analysis software (NIH). The infarct size was quantified by using the formula, infarct volume = {(volume of contralateral hemisphere) – (volume of non-ischemic ipsilateral hemisphere)}/volume of contralateral hemisphere. This formula accounts for the possible interference of brain edema on infarct volume.

### Gelatin Zymography

Tissue lysates of untreated and treated ipsilateral brain hemispheres of MCAO subjected rats as well as the respective brain tissue of sham controls with equal amounts of protein were subjected to 10% polyacrylamide gels that were co-polymerized with substrate (2 mg/mL gelatin) at 4°C. The proteins were renatured by washing the gel twice (each time for 30 min) with 2.5% Triton X-100 and then incubated in substrate buffer (50 mM Tris–HCl, 5 mM CaCl_2_, pH 7.6) for 48 h at 37°C. After a rinse in water, the gel was stained with amido black and destained for appropriate color contrast.

### Terminal deoxy nucleotidyl transferase-mediated nick end labeling (TUNEL) assay

The extent of apoptosis in the ischemic core and the penumbra regions of paraffin-embedded coronal brain sections of animals from all the groups were analyzed by TUNEL assay using an *In Situ* Cell Death Detection Kit (Roche, Indianapolis, IN) according to the manufacturer's instructions. Briefly, the paraffin-embedded tissue sections were de-paraffinized, re-hydrated, treated with proteinase K working solution, and permeabilized. Permeabilized tissue sections were incubated with the TUNEL reaction mixture in a humidified atmosphere for 60 minutes at 37°C in the dark. Sections were counterstained for nuclei with DAPI (Dako, Carpinteria, CA), cover slipped using fluorescent mounting medium (Dako), and observed under a fluorescence microscope (Olympus IX71).

### Statistical analysis

Results are expressed as the mean ± SEM. Statistical comparisons were performed using Graph Pad Prism software (version 3.02). Quantitative data either from TTC staining or TUNEL assay, which have only two groups to compare was evaluated for statistical significance by Unpaired t Test. Quantified data of immunoblots, gelatin zymogram, and immunohistochemistry were evaluated for statistical significance by one-way ANOVA followed by Bonferroni's Multiple Comparison Test. Differences in the values were considered significant at *p* < 0.05.

## References

[b1] Banerjee S., Williamson D., Habib N., Gordon M. & Chataway J. Human stem cell therapy in ischaemic stroke: a review. Age Ageing 40, 7–13 (2011). 21071454 10.1093/ageing/afq133

[b2] del Zoppo G. J. tPA: a neuron buster, too? Nat. Med. 4, 148–150 (1998). 9461181 10.1038/nm0298-148

[b3] Hacke W. *et al.* Thrombolysis in acute ischemic stroke: controlled trials and clinical experience. Neurology 53, S3–14 (1999). 10532643

[b4] Wang X. *et al.* Lipoprotein receptor-mediated induction of matrix metalloproteinase by tissue plasminogen activator. Nat. Med. 9, 1313–1317 (2003). 12960961 10.1038/nm926

[b5] Gasche Y. *et al.* Early appearance of activated matrix metalloproteinase-9 after focal cerebral ischemia in mice: a possible role in blood-brain barrier dysfunction. J. Cereb. Blood Flow Metab. 19, 1020–1028 (1999). 10478654 10.1097/00004647-199909000-00010

[b6] Heo J. H. *et al.* Matrix metalloproteinases increase very early during experimental focal cerebral ischemia. J. Cereb. Blood Flow Metab. 19, 624–633 (1999). 10366192 10.1097/00004647-199906000-00005

[b7] Mun-Bryce S. & Rosenberg G. A. Matrix metalloproteinases in cerebrovascular disease. J. Cereb. Blood Flow Metab. 18, 1163–1172 (1998). 9809504 10.1097/00004647-199811000-00001

[b8] Asahi M. *et al.* Effects of matrix metalloproteinase-9 gene knock-out on the proteolysis of blood-brain barrier and white matter components after cerebral ischemia. J. Neurosci. 21, 7724–7732 (2001). 11567062 10.1523/JNEUROSCI.21-19-07724.2001 PMC6762894

[b9] Lo E. H., Dalkara T. & Moskowitz M. A. Mechanisms, challenges and opportunities in stroke. Nat. Rev. Neurosci. 4, 399–415 (2003). 12728267 10.1038/nrn1106

[b10] Lo E. H., Broderick J. P. & Moskowitz M. A. tPA and proteolysis in the neurovascular unit. Stroke 35, 354–356 (2004). 14757877 10.1161/01.STR.0000115164.80010.8A

[b11] Pfefferkorn T. & Rosenberg G. A. Closure of the blood-brain barrier by matrix metalloproteinase inhibition reduces rtPA-mediated mortality in cerebral ischemia with delayed reperfusion. Stroke 34, 2025–2030 (2003). 12855824 10.1161/01.STR.0000083051.93319.28

[b12] Lee S. R. & Lo E. H. Induction of caspase-mediated cell death by matrix metalloproteinases in cerebral endothelial cells after hypoxia-reoxygenation. J. Cereb. Blood Flow Metab. 24, 720–727 (2004). 15241180 10.1097/01.WCB.0000122747.72175.47

[b13] Gu Z. *et al.* A highly specific inhibitor of matrix metalloproteinase-9 rescues laminin from proteolysis and neurons from apoptosis in transient focal cerebral ischemia. J. Neurosci. 25, 6401–6408 (2005). 16000631 10.1523/JNEUROSCI.1563-05.2005 PMC6725288

[b14] Matsumoto S. *et al.* Expression and localization of matrix metalloproteinase-12 in the aorta of cholesterol-fed rabbits: relationship to lesion development. Am. J. Pathol. 153, 109–119 (1998). 9665471 10.1016/s0002-9440(10)65551-4 PMC1852935

[b15] Chandler S., Cossins J., Lury J. & Wells G. Macrophage metalloelastase degrades matrix and myelin proteins and processes a tumour necrosis factor-alpha fusion protein. Biochem. Biophys. Res. Commun. 228, 421–429 (1996). 8920930 10.1006/bbrc.1996.1677

[b16] Belaaouaj A. A., Li A., Wun T. C., Welgus H. G. & Shapiro S. D. Matrix metalloproteinases cleave tissue factor pathway inhibitor. Effects on coagulation. J. Biol. Chem. 275, 27123–27128 (2000). 10859319 10.1074/jbc.M004218200

[b17] Dong Z., Kumar R., Yang X. & Fidler I. J. Macrophage-derived metalloelastase is responsible for the generation of angiostatin in Lewis lung carcinoma. Cell 88, 801–810 (1997). 9118223 10.1016/s0092-8674(00)81926-1

[b18] Cornelius L. A. *et al.* Matrix metalloproteinases generate angiostatin: effects on neovascularization. J. Immunol. 161, 6845–6852 (1998). 9862716

[b19] Dwivedi A. & George S. MMP-12 is important for VSMC proliferation and migration: role of B-catenin signalling. Vascul Pharmacol 45, e129 (2006).

[b20] Wells J. E. *et al.* Matrix metalloproteinase (MMP)-12 expression has a negative impact on sensorimotor function following intracerebral haemorrhage in mice. Eur. J. Neurosci. 21, 187–196 (2005). 15654856 10.1111/j.1460-9568.2004.03829.x

[b21] Svedin P., Hagberg H. & Mallard C. Expression of MMP-12 after neonatal hypoxic-ischemic brain injury in mice. Dev. Neurosci. 31, 427–436 (2009). 19672072 10.1159/000232561

[b22] Romanic A. M., White R. F., Arleth A. J., Ohlstein E. H. & Barone F. C. Matrix metalloproteinase expression increases after cerebral focal ischemia in rats: inhibition of matrix metalloproteinase-9 reduces infarct size. Stroke 29, 1020–1030 (1998). 9596253 10.1161/01.str.29.5.1020

[b23] Montaner J. *et al.* Matrix metalloproteinase expression after human cardioembolic stroke: temporal profile and relation to neurological impairment. Stroke 32, 1759–1766 (2001). 11486102 10.1161/01.str.32.8.1759

[b24] Svedin P., Hagberg H., Savman K., Zhu C. & Mallard C. Matrix metalloproteinase-9 gene knock-out protects the immature brain after cerebral hypoxia-ischemia. J. Neurosci. 27, 1511–1518 (2007). 17301159 10.1523/JNEUROSCI.4391-06.2007 PMC6673738

[b25] Hu Q. *et al.* Therapeutic application of gene silencing MMP-9 in a middle cerebral artery occlusion-induced focal ischemia rat model. Exp. Neurol. 216, 35–46 (2009). 19073180 10.1016/j.expneurol.2008.11.007

[b26] Bonoiu A. *et al.* MMP-9 gene silencing by a quantum dot-siRNA nanoplex delivery to maintain the integrity of the blood brain barrier. Brain Res. 1282, 142–155 (2009). 19477169 10.1016/j.brainres.2009.05.047 PMC2762384

[b27] Hu Q. *et al.* Lentivirus-mediated transfer of MMP-9 shRNA provides neuroprotection following focal ischemic brain injury in rats. Brain Res. 1367, 347–359 (2011). 20950592 10.1016/j.brainres.2010.10.002

[b28] Mahajan S. D. *et al.* Suppression of MMP-9 expression in brain microvascular endothelial cells (BMVEC) using a gold nanorod (GNR)-siRNA nanoplex. Immunol. Invest 41, 337–355 (2012). 21864113 10.3109/08820139.2011.604863

[b29] Power C. *et al.* Intracerebral hemorrhage induces macrophage activation and matrix metalloproteinases. Ann. Neurol. 53, 731–742 (2003). 12783419 10.1002/ana.10553

[b30] Wasserman J. K., Zhu X. & Schlichter L. C. Evolution of the inflammatory response in the brain following intracerebral hemorrhage and effects of delayed minocycline treatment. Brain Res. 1180, 140–154 (2007). 17919462 10.1016/j.brainres.2007.08.058

[b31] Gronski T. J. Jr *et al.* Hydrolysis of a broad spectrum of extracellular matrix proteins by human macrophage elastase. J. Biol. Chem. 272, 12189–12194 (1997). 9115292 10.1074/jbc.272.18.12189

[b32] Liu Y. *et al.* Matrix metalloproteinase-12 contributes to neuroinflammation in the aged brain. Neurobiol. Aging 34, 1231–1239 (2013). 23159549 10.1016/j.neurobiolaging.2012.10.015

[b33] Yong V. W., Power C., Forsyth P. & Edwards D. R. Metalloproteinases in biology and pathology of the nervous system. Nat. Rev. Neurosci. 2, 502–511 (2001). 11433375 10.1038/35081571 PMC7097548

[b34] Churg A. *et al.* Acute cigarette smoke-induced connective tissue breakdown requires both neutrophils and macrophage metalloelastase in mice. Am. J. Respir. Cell Mol. Biol. 27, 368–374 (2002). 12204900 10.1165/rcmb.4791

[b35] Larsen P. H. & Yong V. W. The expression of matrix metalloproteinase-12 by oligodendrocytes regulates their maturation and morphological differentiation. J. Neurosci. 24, 7597–7603 (2004). 15342725 10.1523/JNEUROSCI.2092-04.2004 PMC6729624

[b36] Larsen P. H., DaSilva A. G., Conant K. & Yong V. W. Myelin formation during development of the CNS is delayed in matrix metalloproteinase-9 and -12 null mice. J. Neurosci. 26, 2207–2214 (2006). 16495447 10.1523/JNEUROSCI.1880-05.2006 PMC6674803

[b37] Vos C. M., van Haastert E. S., de Groot C. J., van der Valk P. & de Vries H. E. Matrix metalloproteinase-12 is expressed in phagocytotic macrophages in active multiple sclerosis lesions. J. Neuroimmunol. 138, 106–114 (2003). 12742660 10.1016/s0165-5728(03)00036-5

[b38] Ulrich R. *et al.* MMP-12, MMP-3, and TIMP-1 are markedly upregulated in chronic demyelinating theiler murine encephalomyelitis. J. Neuropathol. Exp. Neurol. 65, 783–793 (2006). 16896312 10.1097/01.jnen.0000229990.32795.0d

[b39] Lanone S. *et al.* Overlapping and enzyme-specific contributions of matrix metalloproteinases-9 and -12 in IL-13-induced inflammation and remodeling. J. Clin. Invest 110, 463–474 (2002). 12189240 10.1172/JCI14136 PMC150413

[b40] Chelluboina B., Klopfenstein J. D., Gujrati M., Rao J. S. & Veeravalli K. K. Temporal regulation of apoptotic and anti-apoptotic molecules after middle cerebral artery occlusion followed by reperfusion. Mol. Neurobiol. 49, 50–65 (2014). 23813097 10.1007/s12035-013-8486-7 PMC3918127

[b41] Veeravalli K. K. *et al.* Human umbilical cord blood stem cells upregulate matrix metalloproteinase-2 in rats after spinal cord injury. Neurobiol. Dis. 36, 200–212 (2009). 19631747 10.1016/j.nbd.2009.07.012

